# Low-Dose Radiation Affects Cardiovascular Disease Risk in Human Aortic Endothelial Cells by Altering Gene Expression under Normal and Diabetic Conditions

**DOI:** 10.3390/ijms23158577

**Published:** 2022-08-02

**Authors:** Soo-Ho Lee, Ye Ji Jeong, Jeongwoo Park, Hyun-Yong Kim, Yeonghoon Son, Kwang Seok Kim, Hae-June Lee

**Affiliations:** 1 Divisions of Radiation Biomedical Research, Korea Institute of Radiological and Medical Sciences, Seoul 01812, Korea; skarn88@naver.com (S.-H.L.); brightwisdm0914@gmail.com (Y.J.J.); jwpark@kmedihub.re.kr (J.P.); khy9514@nate.com (H.-Y.K.); sonyh@kirams.re.kr (Y.S.); 2 New Drug Development Center, Daegu Gyeongbuk Medical Innovation Foundation, Daegu 41061, Korea

**Keywords:** low-dose radiation, endothelial cells, diabetes mellitus, gene profiling, cardiovascular disease

## Abstract

High doses of ionizing radiation can cause cardiovascular diseases (CVDs); however, the effects of <100 mGy radiation on CVD remain underreported. Endothelial cells (ECs) play major roles in cardiovascular health and disease, and their function is reduced by stimuli such as chronic disease, metabolic disorders, and smoking. However, whether exposure to low-dose radiation results in the disruption of similar molecular mechanisms in ECs under diabetic and non-diabetic states remains largely unknown; we aimed to address this gap in knowledge through the molecular and functional characterization of primary human aortic endothelial cells (HAECs) derived from patients with type 2 diabetes (T2D-HAECs) and normal HAECs in response to low-dose radiation. To address these limitations, we performed RNA sequencing on HAECs and T2D-HAECs following exposure to 100 mGy of ionizing radiation and examined the transcriptome changes associated with the low-dose radiation. Compared with that in the non-irradiation group, low-dose irradiation induced 243 differentially expressed genes (DEGs) (133 down-regulated and 110 up-regulated) in HAECs and 378 DEGs (195 down-regulated and 183 up-regulated) in T2D-HAECs. We also discovered a significant association between the DEGs and the interferon (IFN)-I signaling pathway, which is associated with CVD by Kyoto Encyclopedia of Genes and Genomes (KEGG) pathway analysis, protein–protein network analysis, and module analysis. Our findings demonstrate the potential impact of low-dose radiation on EC functions that are related to the risk of CVD.

## 1. Introduction

With industrial and scientific developments, the risk of radiation exposure has increased in all parts of our lives. Issues such as occupational radiation exposure, the future of nuclear power, manned space flights, and the threat of radiological terrorism call for a thorough understanding of the health risks associated with low-dose radiation exposure [1]. People also experience low-dose radiation exposure from the medical use of radiation for diagnostic purposes. According to the International Commission on Radiological Protection (ICRP) recommendation, those at risk for repeated radiation exposure include health care and nuclear industry workers, who are typically monitored and restricted to effective doses of 100 mSv every five years (i.e., 20 mSv per year), with a maximum of 50 mSv allowed in any given year [2,3]. In contrast, radiation exposure in patients who commonly undergo multiple medical imaging procedures is not typically monitored [4]. While experimental and epidemiologic evidence has linked exposure to low-dose ionizing radiation with the development of solid cancers and leukemia, the association between long-term risk of cardiovascular disease (CVD) and low-dose radiation exposure is unknown [5]. Since the biological mechanisms underlying CVD after low-dose are unclear and results from epidemiological studies are inconsistent, only a weak relationship between CVD and low-dose radiation exposure has been reported. Therefore, molecular studies should be conducted to improve our understanding of the pathogenesis and risk estimation of radiation-induced CVD at low doses.

Endothelial cells (ECs) are a key component of the cardiovascular system, and their function is diminished by the presence of cardiovascular risk factors. Several stimuli, including chronic disease states, metabolic conditions (e.g., type 2 diabetes mellitus (T2DM), obesity, dyslipidemia), smoking, and disturbed blood flow drive EC dysfunction [6]. There is a particularly close link between T2DM and CVD. Progression of T2DM eventually involves the development of chronic vascular impairment, which results in CVD. These cardiovascular complications are the main causes of death in patients with diabetes mellitus (DM) worldwide [7,8]. It has been reported that diabetes emerged as a late effect of radiation therapy among childhood cancer survivors who underwent total body irradiation or radiation to the head and the abdomen [9,10,11,12,13,14,15]. In addition, based on epidemiological studies of A-bomb survivors, an association between radiation and the incidence of diabetes was hypothesized [16]. Interestingly, the therapeutic effect of repeated low-dose radiation (25 or 50 mGy) on diabetes-induced cardiopathy has been reported; low-dose radiation ameliorated DM-induced cardiopathy caused by anti-oxidant exposure [17] and prevented cardiac inflammation and pathological remodeling [18]. The major mechanisms of action and therapeutic potential of low-dose radiation on chemical- or radiation-induced tissue damage were hypothesized to include the induction of hormesis and adaptive responses [19], suggesting that low-dose radiation may promote different responses in healthy or diseased conditions. The risk of disease rises over time as the number of metabolic syndrome characteristics increases; therefore, early intervention is warranted.

In this study, we investigated the effect of low-dose radiation on EC function and pathophysiology using RNA sequencing (RNA-seq) to identify its molecular mechanisms using primary HAECs derived from healthy or T2DM donors. This study provides valuable insights into the effects of combining low-dose radiation with other disease risk factors, including metabolic syndrome and DM, on CVD.

## 2. Results

### 2.1. Human Aortic Endothelial Cell Function Was Impaired in Type 2 Diabetes Mellitus Compared to That in Normal Conditions

**Figure 1:**
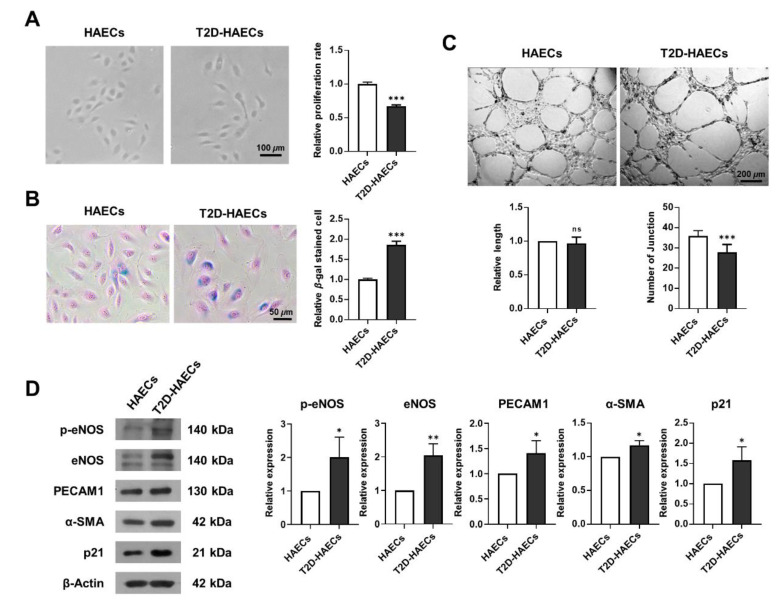
Characterization of functions of primary human aortic endothelial cells (HAECs) and human aortic endothelial cells, diabetes type II (T2D-HAECs). (**A**) Representative images of cultured HAECs and T2D-HAECs. The proliferative rates of HAECs and T2D-HAECs were measured using an MTT assay (n = 4). (**B**) β-galactose staining and quantification in HAECs and T2D-HAECs. Blue represents β-galactose-positive staining (n = 3). (**C**) Images of tube formation in HAECs and T2D-HAECs 16 h after seeding of 1.5 × 10^4^ cells/100 μL. The length and number of tube junctions were counted and compared in each group (n = 6). (**D**) Western blotting of endothelial cell function-related proteins (n = 3). Quantification of each protein is shown in the graph. All data represent the mean ± standard deviation (SD) of each independent experiment; * *p* < 0.05; ** *p* < 0.01; *** *p* < 0.005; ns = no significance.

We assessed whether human aortic endothelial cells, diabetes type II (T2D)-HAECs exhibited dysfunctional phenotypes when compared to normal HAECs (HAECs). DM induces endothelial dysfunction [20]; therefore, we examined endothelial proliferation rates and tube formation in HAECs and T2D-HAECs. T2D-HAECs showed slower proliferation and an increased number of β-galactose-positive cells when compared to normal HAECs (Figure 1A,B). Additionally, T2D-HAECs showed fewer tubes than normal HAECs (Figure 1C). Western blotting assays revealed that T2D-HAECs showed increased p-eNOS [21], PECAM1 [22], α-SMA [23], and p21 [24] expression compared to normal HAECs, indicating impaired endothelial function (Figure 1D). 

**Figure 2:**
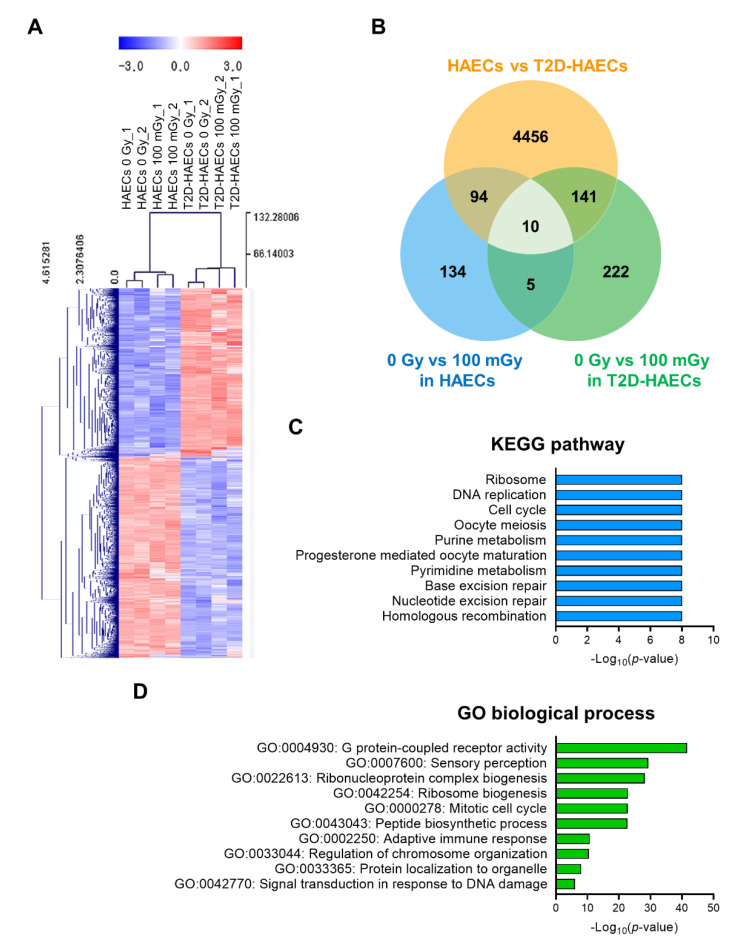
Analysis of differentially expressed genes (DEGs) in T2D-HAECs and normal HAECs. (**A**) Heatmap analysis with hierarchical clustering of DEGs in T2D-HAECs compared to normal HAECs. (**B**) Venn diagram of the comparisons between HAECs and T2D-HAECs following treatment with or without low-dose radiation. (**C**) The 10 most significantly altered pathways associated with the identified DEGs, according to KEGG analysis (*p* < 0.05, FDR *q*-value < 0.25). (**D**) Top 10 significant GO terms of biological processes associated with the identified DEGs.

We performed RNA-seq analysis using total RNA isolated from HAECs and T2D-HAECs treated with or without 100 mGy of ionizing radiation. Whole transcriptome analysis showed that T2D affected the normal EC transcriptome and that this T2D-induced transcriptional alteration was greater than that in ECs treated with 100 mGy of ionizing radiation. To confirm the EC gene signatures affected by T2DM, we analyzed the gene profiles of T2D-HAECs compared to those of HAECs. A total of 27,685 genes were analyzed; 4701 differentially expressed genes (DEGs) with *p*-values < 0.05 and fold changes > 1.2 were found (Table S1). These DEGs were represented in a heatmap with hierarchical clustering (Figure 2A). Kyoto Encyclopedia of Genes and Genomes (KEGG) analysis showed that T2DM in ECs most significantly altered the pathways associated with the ribosome, DNA replication, cell cycle, oocyte meiosis, and purine metabolism (Figure 2C, Table S2). Gene ontology (GO) analysis of biological processes showed that the DEGs were most significantly involved in G-protein-coupled receptor activity, sensory perception, ribonucleoprotein complex biogenesis, ribosome biogenesis, and the mitotic cell cycle in T2D-HAECs (Figure 2D, Table S3).

### 2.2. Gene Profiling of HAECs after 100 mGy Ionizing Radiation Treatment

**Figure 3:**
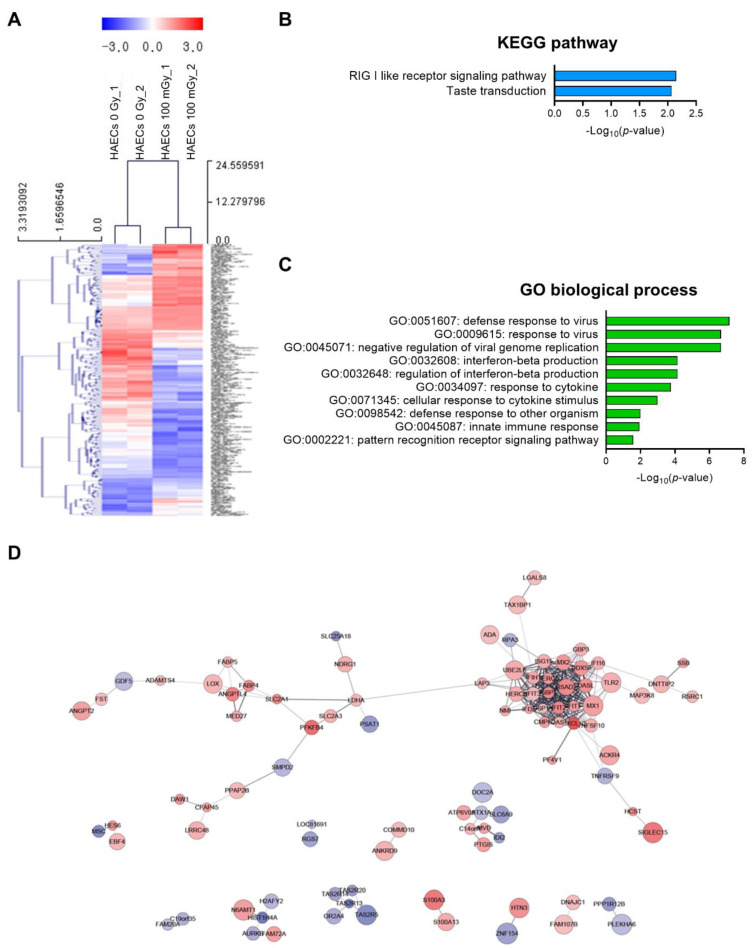
DEG analysis of low-dose radiation-related DEGs in normal HAECs. (**A**) Heatmap analysis with hierarchical clustering of DEGs in HAECs treated with and without low-dose radiation. (**B**) Significant KEGG functional pathways associated with the identified DEGs in HAECs treated with and without low-dose radiation (*p* < 0.05, FDR *q*-value < 0.25). (**C**) Significant GO terms of biological processes associated with the DEGs identified in HAECs treated with and without low-dose radiation (*p* < 0.05). (**D**) The protein–protein interaction (PPI) networks in HAECs treated with and without low-dose radiation were visualized using the STRING plug-in of the Cytoscape program (version 3.9.1). Node colors represent the expression. The gradual change in color from blue to red corresponds to the change in the expression level (from down-regulation to up-regulation) in low-dose radiation-treated HAECs versus non-treated HAECs.

Next, we analyzed low-dose radiation-responsive genes in normal HAECs; results revealed 243 DEGs with a significance threshold of *p*-value < 0.05 and fold change >1.2. When compared with that in untreated cells, 110 DEGs were up-regulated and 133 were down-regulated after irradiation (Table S4). The 20 most altered genes by fold change are listed in Table 1. A heatmap analysis with hierarchical clustering of DEGs responsive to low-dose radiation in HAECs is shown in Figure 3A. KEGG pathway analysis showed that DEGs involved in the RIG-I-like receptor signaling pathway and taste transduction were most significantly altered in HAECs exposed to 100 mGy of radiation (Figure 3B) and involved in GO terms associated with viral infection and innate immune-related biological processes (Figure 3C). Protein–protein interaction (PPI) network analysis indicated proteins from 100 mGy-irradiated normal HAECs, represented by 186 nodes and 293 edges between 243 DEGs (Figure 3D). To investigate the interactions between PPI networks, functional enrichment analysis was performed; results revealed that PPI networks from 100 mGy-irradiated normal HAECs involved interferon (IFN) alpha/beta signaling, negative regulators of DDX58/IFIH1 signaling, and ISG15-protein conjugation. Together, these results showed that low-dose radiation could influence transcriptomes and HAEC signaling pathways.

### 2.3. Gene Profiling in T2D-HAECs after Treatment with 100 mGy of Ionizing Radiation

**Figure 4:**
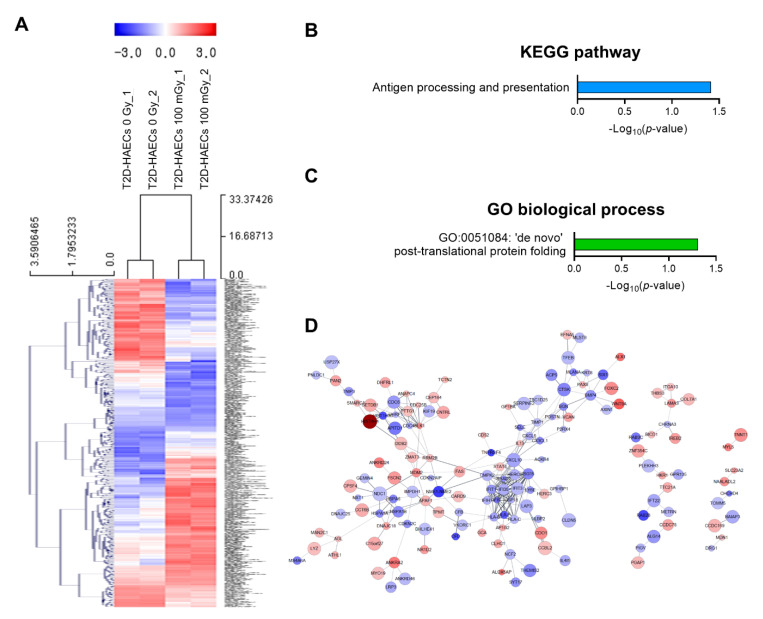
DEG analysis of low-dose radiation-related DEGs in T2D-HAECs. (**A**) Heatmap analysis with hierarchical clustering of DEGs in T2D-HAECs treated with 100 mGy irradiation. (**B**) Significant KEGG functional pathways related to DEGs in T2D-HAECs treated with 100 mGy irradiation (*p* < 0.05, FDR *q*-value < 0.25). (**C**) Significant GO term of associated biological processes in DEGs in T2D-HAECs treated with 100 mGy irradiation (*p* < 0.05). (**D**) The PPI networks associated with low-dose radiation responsiveness in T2D-HAECs visualized using the STRING plug-in of the Cytoscape program (version 3.9.1). Node colors represent the expression. The gradual change in color from blue to red corresponds to the change in the expression level (from down-regulation to up-regulation) in low-dose radiation-treated HAECs versus non-treated HAECs.

We also analyzed low-dose radiation-induced genes in human ECs in a diabetic state. We identified 378 DEGs with a significance threshold of *p*-value < 0.05 and fold change > 1.2, as shown in the heatmap in Figure 4A. When T2D-HAECs were treated with 100 mGy of ionizing radiation, 183 DEGs were up-regulated and 195 were down-regulated when compared to that in untreated cells (Table S5). The 20 most significantly modulated genes by fold change are listed in Table 2. KEGG pathway analysis of T2D-HAECs showed that low-dose radiation altered a pathway involved in antigen processing and presentation (Figure 4B), while the GO term for post-translational protein folding was also identified in these cells (Figure 4C). PPI networks identified possible contacts between proteins encoded by 378 DEGs that contained 284 nodes and 291 edges in 100 mGy-irradiated T2D-HAECs (Figure 4D). 

### 2.4. Regulation of Cell Function Following Response to Low-Dose Radiation in ECs under Normal and Diabetic Conditions

**Figure 5:**
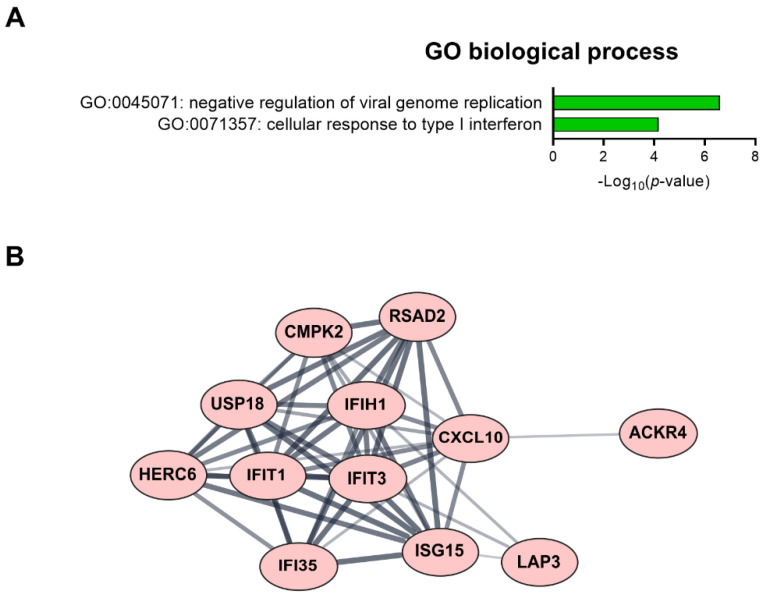
Fifteen candidate genes showing significantly modulated expression following low-dose radiation in both HAECs and T2D-HAECs. (**A**) Significant GO terms of biological process associated with the identified DEGs. (**B**) PPI network analysis identified the gene hub involved in the interferon signaling pathway.

**Figure 6:**
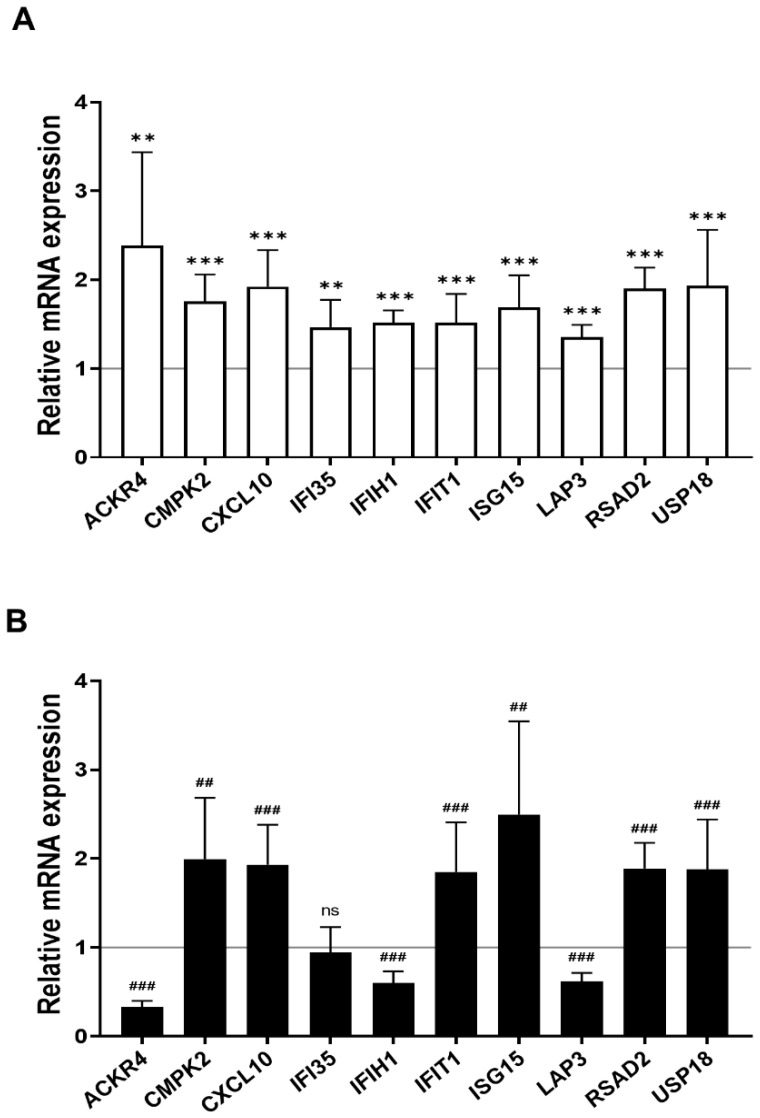
Quantitative PCR validation of common low-dose radiation-responsive genes expressed in HAECs and T2D-HAECs. Values are represented as mean ± SD (n = 6). (A) Related gene expression of normal HAECs by 100 mGy radiation, ** *p* < 0.01, *** *p* < 0.005 vs. non-irradiated HAECs by Student’s *t*-test was considered statistically significant. (B) Related gene expression of T2D-HAECs by 100 mGy radiation, ^##^ *p* < 0.01, ^###^ *p* < 0.005 vs. non-irradiated T2D-HAECs by Student’s *t*-test was considered statistically significant. ns = no significance.

**Figure 7:**
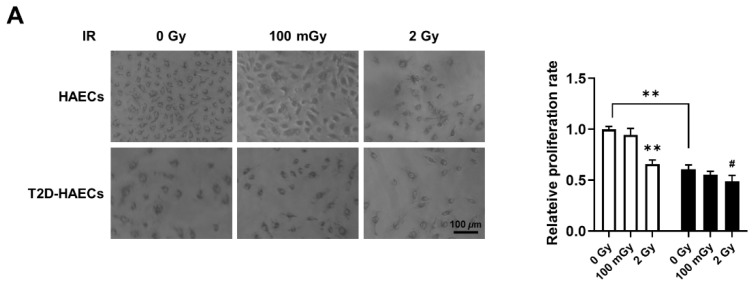

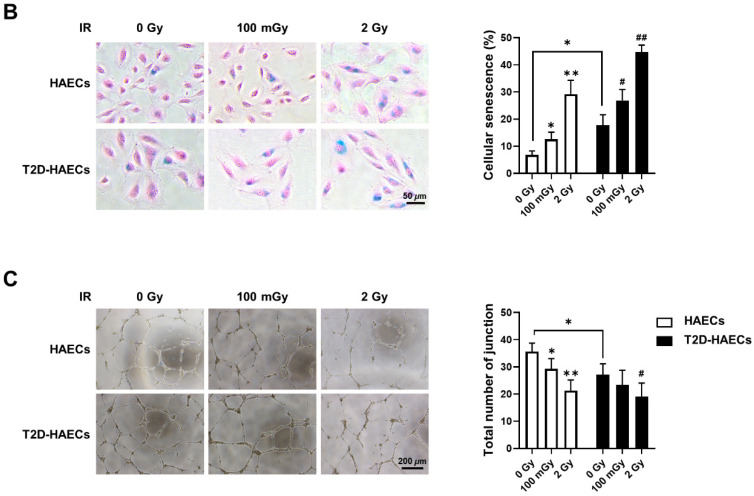
Evaluation of cell proliferation rate, cellular senescence, and tube formation in HAECs and T2D-HAECs after 100 mGy or 2 Gy of ionizing radiation treatments. (**A**) Representative images of cultured HAECs and T2D-HAECs. The proliferative rate of HAECs and T2D-HAECs was measured using an MTT assay (n = 4). (**B**) β-galactose staining and quantification in HAECs and T2D-HAECs. Blue represents β-galactose-positive staining (n = 3). (**C**) Images of tube formation in HAECs and T2D-HAECs 16 h after seeding 1.5 × 10^4^ cells/100 μL. The length and the number of junctions in the tubes were counted and compared in each group (n = 4). All data represent the mean ± SD of three or four independent experiments; * *p* < 0.05, ** *p* < 0.01 vs. non-irradiated HAECs. ^#^ *p* < 0.05, ^##^ *p* < 0.01 vs. non-irradiated T2D-HAECs.

We found 15 common DEGs that were significantly altered in both HAECs and T2D-HAECs after exposure to low-dose radiation (Table 3). No KEGG pathways were identified for these DEGs; however, 15 DEGs were related to two GO terms associated with the negative regulation of viral genome replication and cellular response to type I IFN (Figure 5A). The PPI network analysis showed that 12 of these 15 DEGs closely interacted (Figure 5B). We validated gene sets in both HAECs and T2D-HAECs treated with 100 mGy irradiation by qRT-PCR (Figure 6). Consistent with the RNA-seq results, *ACKR4*, *IFIH1*, and *LAP3* were up-regulated in HAECs but down-regulated in T2D-HAECs. However, the mRNA expression of *CMPK2*, *CXCL10*, *IFI35*, *IFT1*, *ISG15*, *RSAD2*, and *USP18* was up-regulated in HAECs after low-dose radiation, while that of *CMPK2*, *CXCL10*, *IFT1*, *ISG15*, *RSAD2*, and *USP18* was up-regulated in T2D-HAEC, which was not consistent with the RNA-seq results. The other genes (*ASTE1, IFIT3, HERC6, TASR13,* and *TAS2R20*) were not altered. We examined the cell proliferation rate, cellular senescence, and tube formation activity in HAECs and T2D-HAECs treated with 100 mGy and 2 Gy of radiation and found that low-dose radiation increased the number of β-galactose-positive cells and reduced tube formation activity, similar to that seen in non-irradiated T2D-HAECs (Figure 7). However, both cell types showed the same responses to high-dose radiation exposure, indicated by their lower proliferative rates, increased cellular senescence, and reduced tube formation compared with non-irradiated HAECs or T2D-HAECs, respectively.

## 3. Discussion

Although public concern about low-dose radiation exposure has increased with technological development and the increased application of radiation technology in the fields of medicine and industry, clinically relevant tissue damage does not occur below an absorbed dose of 100 mGy; this forms the basis of current radioprotection systems against non-cancer effects. However, recent epidemiological findings point to a risk of non-cancerous disease after exposure to lower doses of ionizing radiation than previously thought, especially for CVD and cataracts [25]. Owing to limited statistical support, the relationship between dose and risk is undecided below 0.5 Gy [26]. However, emerging evidence suggests that doses under 0.5 Gy may increase the long-term risk of CVD [27]. Moreover, environmental factors, including radiation, can accelerate the onset and progression of diabetes with more serious side effects [28]. Similar to diabetes, radiation causes damage to blood vessels and tissues and affects aging [29]. Extensive clinical and experimental evidence suggests that endothelial dysfunction occurs in T2DM and prediabetes patients [20]. Endothelial dysfunction has received increased attention as a potential contributor to the pathogenesis of vascular disease in T2DM [30]. 

In this study, we used gene profiling analysis to investigate the possible impact of low-dose radiation on the induction of endothelial dysfunction. Impairment of endothelial cell function is a critical risk factor for both cardiovascular pathology and DM. Thus, we analyzed low-dose radiation-responsive gene profiles in irradiated ECs under normal and DM conditions. We performed RNA-seq using total RNA from HAECs and T2D-HAECs exposed to 100 mGy of low-dose radiation and profiled genes that may be associated with endothelial damage and dysfunction that cause CVD. We confirmed that T2D-HAECs exhibited impaired EC characteristics and gene profiles that were strikingly different from those of HAECs. We observed that low-dose radiation of normal ECs promoted a phenotype similar to that of non-irradiated diabetes, which showed increased numbers of β-galactose-positive cells and reduced tube formation, further indicating impaired EC function. Additionally, 2 Gy of high-dose radiation induced more significant EC dysfunction, including reduced proliferation rate, cellular senescence, and tube formation in both HAECs and T2D-HAECs when compared to those of the controls. However, in T2D-HAECs, low-dose radiation did not induce significant changes in EC function. Indeed, similar to the EC functional assessment, HAECs showed more consistent results in qRT-PCR validation assays of the RNA-seq results compared with T2D-HAECs. qRT-PCR analysis showed that low-dose irradiation of T2D-HAECs resulted in only three reproducible gene products compared to that with RNA-seq results. Similar to the results of EC functional assessments, low-dose radiation had no significant effect on the qRT-PCR outcome. These different responses between HAECs and T2D-HAECs to low-dose radiation may be caused by inadequate radiation to phenotypically or mechanistically alter HAECs under diabetic conditions. Our findings were similar to those of Vieria Dias et al., who investigated the response of HAECs to different low-dose (6 mGy/h) and high-dose (1 Gy/min) rates of 0.05, 0.5, 1.0, and 2 Gy [31]. They reported that the threshold for angiogenic capacity loss in cells treated with both low- and high-dose rates of radiation was between 0.5 and 1.0 Gy [31]. However, the effect of 100 mGy under diabetic conditions has not been investigated. Zhang et al. tested whether 14 repeated exposures of 12.5, 25, or 50 mGy every two days for two weeks could protect streptozotocin-induced diabetic C57BL/6J mice against diabetes-induced cardiopathy and reported that only repeated 25 and 50 mGy exposures exerted protective effects in the form of not only anti-apoptotic and anti-oxidant effects but also in preventing cardiomyocyte hypertrophy and fibrosis [17]. However, Zhang et al. reported that irradiating mice with doses higher than 100 mGy did not show any significant effects on diabetes-induced cardiopathy, including anti-oxidant enzyme levels or histopathological alterations to the heart [17]. Takahashi et al. reported that among acute exposures to doses of 0.25, 0.5, 1.0, and 2.0 Gy, only exposure to acute doses of 0.5 Gy provided significant protection against ALX-induced diabetes, while 0.25 Gy was not adequate to exert a protective effect against the development of diabetes [32]. While it has been reported that low-dose radiation induced damage in normal ECs in the form of morphological changes, proliferation, and DNA damage [31,33], no studies have examined the effect of low-dose radiation (<100 mGy) on ECs under diabetic conditions; thus, further studies are needed to estimate the risk of CVD by providing evidence of the biological mechanism involved in its pathogenesis. 

CVD and T2DM share several common pathophysiological features, including insulin resistance, inflammation, oxidative stress, hypercoagulability, high blood pressure, dyslipidemia, and obesity [34]. We investigated the radiation-induced pathogenic mechanism linked to CVD and diabetes using bioinformatic analysis. Examining the impact of low-dose radiation on EC dysfunction indicated potential CVD risk, while PPI network analysis revealed a hub of 12 genes (*ACKR4*, *CMPK2*, *CXCL10*, *HERO6*, *IFI35*, *IFIH1*, *IFT1*, *IFT3*, *ISG15*, *LAP3*, *RSAD2*, and *USP18*) out of 15 DEGs that were associated with IFN-I signaling. IFNs constitute a family of cytokines that is divided into three main subtypes: type I, II, and III IFNs. The type I IFNs (IFN-α and IFN-β) are reported to be important mediators in atherosclerosis [35]. Using qRT-PCR, we confirmed that low-dose radiation significantly altered mRNA levels of *ACKR4*, *IFIH1*, *CMPK2*, *CXCL10*, *IFI35*, *IFT1*, *ISG15*, *LAP3*, *RSAD2*, and *USP18*, which are involved in IFN-I signaling. It is known that IFIH1 (interferon-induced helicase-1), IFI35 (interferon-induced protein 35), and USP18 (ubiquitin-specific peptidases 18) activate the IFN signaling pathway; IFIH1 induces pro-inflammatory cytokines, and type I IFNs respond to viral infections in which they act as innate immune receptors [36,37]. It has been suggested that viral infections can cause autoimmune diseases such as type 1 diabetes (T1D) through β-cell disruption [38]; however, this remains debatable. IFIT3 is a novel biomarker for human ischemic cardiomyopathy that can inhibit the nuclear factor-kappa B (NF-κB) pathway in injured arteries, resulting in the inhibition of endothelial cell proliferation, migration, and re-endothelialization [39]. Therefore, IFI35 may induce diverse diseases, including hyperplasia, atherosclerosis, and CVD after endothelial injury [40]. 

USP18 is expressed in the heart, where it modulates inflammation and apoptosis [39]. USP18 is stimulated by IFNs, which form a negative feedback loop and act as isopeptidases for specific substrates with ISG15, a ubiquitin-like protein [41]. ISG15 is also induced by IFNs and can modify proteins in a ubiquitin-like manner. This modification in cardiomyocytes has been found to contribute to the suppression of viral replication in a CVB-3-infected mouse model; therefore, ISG15 modification may be a critical part of the innate immune system in cardiomyocytes [42]. CMPK2 (cytidine/uridine monophosphate kinase 2) is localized in the mitochondria, where it engages in macrophage differentiation that is involved in atherosclerosis processes. Deletion of CMPK2 has been reported to reduce mtROS production induced by IFN-α, suggesting that it exerts pro-atherogenic effects [43]. CXCL10 (C-X-C motif chemokine ligand 10) and RSAD2 (radical S-adenosyl methionine domain containing 2) are highly expressed in IFN-γ and LPS-treated vascular smooth muscle cells, which can induce pro-inflammatory and pro-atherogenic processes [44]. ACKR4 (atypical chemokine receptor 4) belongs to the ACKR subfamily and acts as a chemokine receptor for CCL2, CCL8, CCL13, CCL19, CCL21, and CCL25 [45]. It has been reported that the combination of chemokines and ACKR4 can lead to the recruitment of β-arrestin [46,47,48], which mediates cardioprotective signaling that results in ligand internalization [49]. LAP3 (leucine aminopeptidase 3) has been reported to play a vital role in the pathogenesis of nonalcoholic fatty liver disease (NAFLD), which is a known risk factor for T2DM [50] and is associated with cardiovascular and cerebrovascular diseases [51]. 

Our study has some limitations. Although we discovered unique gene profiles pertaining to key genes involved in IFN-I signaling by low-dose radiation, the up- or down-regulated expression patterns of some key genes were inconsistent with the RNA-seq results in T2D-HAECs. Thus, it is our assumption that the pathophysiological features of diabetes may be more potent than the biological effects exerted by low-dose radiation. Therefore, further studies are required to uncover the biological mechanisms active in normal conditions as well as pathological conditions. Furthermore, we did not perform in vivo experiments to clarify the roles of key molecules via the modulation of gene or protein levels and their corresponding physiological features. Moreover, cultured cells are known to undergo genetic variations, and these modifications may alter the responses to irradiation, which would not be evident in primary tissues. Thus, future studies are required to further validate the available data. 

In this study, we investigated the effect of low-dose radiation on EC gene profiles in HAECs derived from healthy or T2DM donors. We demonstrated that low-dose radiation could reduce the proliferative rate, increase cellular senescence, and reduce tube formation in normal HAECs, which is consistent with results from cells treated with 2 Gy high-dose radiation. Additionally, low-dose radiation induced changes in EC phenotypes compared to those observed in non-irradiated diabetic conditions, indicating impaired EC function. Moreover, we uncovered key molecules involved in the IFN-I signaling pathway that are associated with CVD in cultured primary ECs under both normal and diabetic conditions. Therefore, our study suggests a potential effect of low-dose radiation on vascular physiology in both normal and diseased states. 

## 4. Materials and Methods

### 4.1. Cell Culture

Human aortic endothelial cells (HAECs, #CC-2535) and human aortic endothelial cells, diabetes type II (T2D-HAECs, #CC-2920) were purchased from Lonza Group Ltd. (Walkersville, MD, USA), cultured in endothelial growth medium-2 microvascular medium (Lonza), and incubated at 37 °C in a 5% CO_2_ humidified incubator. 

### 4.2. Irradiation

Cells were exposed to γ-rays with a ^137^Cs laboratory γ-irradiator (LDI-KCCH 137, Seoul, Korea) at a dose of 0.1 Gy (4.8 mGy/min) or 2 Gy (2.26 Gy/min) using a procedure described previously [52].

### 4.3. MTT Assay 

A 3-(4,5-dimethylthiazol-2-yl)-2,5-diphenyltetrazolium bromide (MTT) assay was used to measure cell proliferation after low-dose radiation exposure. Cells were seeded at 5 × 10^3^ cells/well in 4-well cell culture dishes (SPL Life Sciences, Gyeonggi-do, Korea) and incubated at 37 °C and 5% CO_2_. After 24 h, cells were irradiated with 100 mGy and 2 Gy and then incubated for another 3 d. A total of 5 mg MTT powder (Duchefa Biochemie, Haarlem, The Netherlands) was dissolved in 1 mL Dulbecco’s phosphate-buffered saline (DPBS, Welgene, Gyeongsangbuk-do, Korea) and then mixed into the cell culture medium in a 1:10 ratio, followed by incubation for 2 h. The MTT-supplemented culture medium was removed, and dimethyl sulfoxide (DMSO, Sigma-Aldrich, St. Louis, MO, USA) was added to the cultures, followed by incubation for 30 min. A 100 μL aliquot of the solution was pipetted into 96-well cell culture plates (SPL Life Science) and measured at a wavelength of 570 nm using a Multiskan™ FC microplate photometer (Thermo Fisher Scientific, Waltham, MA, USA)

### 4.4. Tube Formation Assay 

Matrigel (354230, Corning, MA, USA) was coated onto 24-well plates at 37 °C for 2 h before cell detachment. HAECs and T2D-HAECs were irradiated with 0, 100 mGy, and 2 Gy and incubated for an additional 24 h. The next day, cells were detached, seeded into culture plates at 1.5 × 10^4^ cells, and incubated for 16 h at 37 °C. Tube formation was analyzed and quantified using a Nikon ECLIPSE microscope (Nikon, Tokyo, Japan) and its associated software.

### 4.5. Senescence-Associated β-Galactosidase (SA-β-gal) Staining

Cells were irradiated and grown to a density of 1 × 10^4^ cells in 35 mm cell culture dishes for 72 h. The cells were then washed twice with DPBS and fixed at room temperature for 10 min with 3.7% (*v*/*v*) formaldehyde. Fixed cells were incubated with an SA-β-galactose mixed solution containing 1 mg/mL 5-bromo-4-chloro-3-indolyl-β-d-galactosidase (Mbiotech, Gyeonggi-do, Korea) in DMSO, 40 mM citric acid (Sigma-Adlrich), 40 mM disodium hydrogen phosphate pH 5.8 (Duchefa Biochemie), 5 mM potassium ferrocyanide, 150 mM sodium chloride, and 2 mM magnesium dichloride for 16 h at 37°C without CO_2_. The stained cells were washed twice with DPBS, counter-stained with 10% eosin solution in double distilled water (DDW) for up to 1 min, and then washed twice with DDW.

### 4.6. Western Blotting 

Total protein was extracted from cell cultures using RIPA lysis buffer (Mbiotech), followed by two washes with DPBS (Welgene) and quantitation via a Bradford assay (Bio-Rad, Hercules, CA, USA) following the manufacturer’s protocol. Equal amounts of total proteins were loaded onto 6–8% SDS–PAGE gels and transferred into NT nitrocellulose membranes (Pall Corporation, Pensacola, FL, USA). Membranes were incubated at 4°C with eNOS (1:1000, sc-634, Santa Cruz Biotechnology, Dallas, TX, USA), p-eNOS (1:1000, #9571s, Cell Signaling Technology, Danvers, MA, USA), p21 (1:1000, sc-6246, Santa Cruz Biotechnology), α-SMA (1:1000, ab5694, Abcam, Cambridge, UK), PECAM-1 (1:1000, ab28364, Abcam), or anti-β-actin (1:3000, #3700, Cell Signaling Technology) in 3% bovine serum albumin (GenDEPOT, Katy, TX, USA). After overnight incubation, the membranes were washed thoroughly in PBS-T buffer and incubated for 1 h with the corresponding secondary antibodies diluted at 1:3000. The proteins were detected using the ECL solution luminescence method (NEL104001EA, PerkinElmer, Waltham, MA, USA).

### 4.7. RNA Isolation

Total RNA was isolated using TRIsure solution (Bioline, London, UK) following the manufacturer’s instructions. RNA quality (expressed as an RNA integrity number) was assessed with an Agilent 2100 bioanalyzer using an RNA 6000 Nano Chip (Agilent Technologies, Amstelveen, The Netherlands). Total RNA was quantified using a NanoDrop 2000 spectrophotometer (ND-2000; Thermo Fisher Scientific, Inc.).

### 4.8. RNA-seq

RNA-seq was performed with high-quality RNA samples (RNA integrity number > 7) isolated from each cell type. Four separate samples were multiplexed into each lane and sequenced on a HiSeq 4000 system (Illumina, San Diego, CA, USA). 

### 4.9. Identification of DEGs and Data Analysis 

The RNA-seq reads were aligned using Bowtie2 [53]. Bowtie2 indices were generated from the genome assembly sequences or the representative transcript sequences for alignment to the genome. The alignment file was used to assemble transcripts, estimate abundance, and detect DEGs. Data mining and graphic visualization were performed using the Excel-based Differentially Expressed Gene Analysis (ExDEGA; Ebiogen Inc., Seoul, Korea). Probe sets without corresponding gene symbols were removed, and sand differences with a *p*-value < 0.05 and absolute fold change > 1.2 were considered statistically significant.

### 4.10. Heatmap Visualization and Hierarchical Clustering Analysis

The expressions of DEGs were visualized as a heatmap using the open-source program MeV (version 4.9.0; https://sourceforge.net/projects/mev-tm4/, accessed on 22 November 2021). Gene expression levels of each sample were indicated by different colors using log_2_ ratio adjusted z-score values, and hierarchical clustering of gene and sample trees was analyzed using the Euclidean distance metric.

### 4.11. Pathway Analysis 

DEG lists were annotated to KEGG pathways to identify significant pathways. KEGG pathways were analyzed using GSEA 4.0.3 software. Pathways with an FDR-adjusted *p*-value < 0.05 were filtered, and a Bonferroni correction was applied to adjust the *p*-values. Pathways were considered significantly enriched if the *p*-value < 0.05 and FDR *q*-value < 0.25. 

### 4.12. Gene Ontology (GO) Analysis 

All DEGs were analyzed by the GO database (http://www.geneontology.org/, accessed on 29 November 2021). Biological processes with an FDR threshold <0.05 were filtered, and biological process-related categories were selected and grouped hierarchically. Cytoscape’s ClueGO plug-in was used to analyze the functional groups of GO terms related to biological processes. Significant interrelated GO terms were determined as adjusted *p*-value < 0.05.

### 4.13. Protein–Protein Interaction Network and Module Analysis

Protein–protein interaction (PPI) networks were mapped and analyzed using Cytoscape (version 3.9.1; https://cytoscape.org/, Gladstone, San Francisco, CA, USA, 22 November 2021). Significant modules in the PPI networks were identified using Molecular Complex Detection (MCODE), a plug-in app of Cytoscape designed to analyze densely connected regions by clustering a given network.

### 4.14. Total RNA Extraction and Quantitative Reverse Transcription-Polymerase Chain Reaction (qRT-PCR)

Total RNA was extracted using the TRIsure solution and reverse-transcribed into cDNA using the SensiFAST^TM^ cDNA Synthesis kit (Bioline) from the isolated RNA. PCR was performed with a Mic Real-Time PCR system (Bio Molecular Systems, QLD, Australia), the Qualitative PCR SYBR 2X Master Mix Kit (Mbiotech), and primer pairs specific for target genes (Table 4).

### 4.15. Statistical Analysis

Data are expressed as mean ± standard deviation (SD). One-way ANOVA followed by the Tukey post hoc analysis or Student’s unpaired two-tailed *t*-test were performed using the GraphPad Prism program (version 8.0, GraphPad Software, San Diego, CA, USA) and are indicated in the figure legends. Values with *p*-values < 0.05 were considered statistically significant.

## Figures and Tables

**Table 1:** List of 20 genes showing the most significantly modulated expression following low-dose radiation in HAECs.

Entrez_ID	Gene Symbol	Description	log_2_(fc) (*p*-Value)
100529097	*RPL36A-HNRNPH2*	RPL36A-HNRNPH2 readthrough	−2.628 (0.034)
5918	*RARRES1*	Retinoic acid receptor responder 1	−0.812 (0.029)
3627	*CXCL10*	C-X-C motif chemokine ligand 10	0.759 (0.014)
255762	*PDZD9*	PDZ domain containing 9	−0.750 (0.040)
257177	*CFAP126*	Cilia and flagella associated protein 126	−0.702 (0.009)
5210	*PFKFB4*	6-phosphofructo-2-kinase/fructose-2,6-biphosPhatase 4	0.658 (0.024)
6781	*STC1*	Stanniocalcin 1	0.627 (0.011)
164668	*APOBEC3H*	Apolipoprotein B mRNA editing enzyme catalytic subunit 3H	−0.612 (0.019)
284266	*SIGLEC15*	Sialic acid binding Ig-like lectin 15	0.602 (0.047)
6274	*S100A3*	S100 calcium binding protein A3	0.597 (0.044)
8359	*HIST1H4A*	Histone cluster 1, H4a	−0.594 (0.012)
50838	*TAS2R13*	Taste 2 receptor member 13	−0.558 (0.002)
116842	*LEAP2*	Liver expressed antimicrobial peptide 2	−0.538 (0.049)
6513	*SLC2A1*	Solute carrier family 2 member 1	0.532 (0.005)
3433	*IFIT2*	Interferon-induced protein with tetratricopeptide repeats 2	0.531 (0.015)
91543	*RSAD2*	Radical S-adenosyl methionine domain containing 2	0.516 (0.031)
2633	*GBP1*	Guanylate binding protein 1	0.509 (0.011)
54429	*TAS2R5*	Taste 2 receptor member 5	−0.507 (0.045)
2167	*FABP4*	Fatty acid binding protein 4	0.501 (0.011)
9242	*MSC*	Musculin	−0.498 (0.015)

**Table 2:** List of 20 genes showing the most significantly modulated expression following low-dose radiation in T2D-HAECs.

Entrez _ID	Gene Symbol	Description	log_2_(fc) (*p*-Value)
100526772	*TMEM110-MUSTN1*	TMEM110-MUSTN1 readthrough	1.504 (0.043)
121504	*HIST4H4*	Histone cluster 4, H4	1.403 (0.050)
90273	*CEACAM21*	Carcinoembryonic antigen-related cell adhesion molecule 21	−0.943 (0.001)
574016	*CLLU1OS*	Chronic lymphocytic leukemia up-regulated 1 opposite strand	−0.927 (0.029)
117608	*ZNF354B*	Zinc finger protein 354B	0.919 (0.016)
654364	*NME1-NME2*	NME1-NME2 readthrough	−0.912 (0.032)
7293	*TNFRSF4*	Tumor necrosis factor receptor superfamily member 4	−0.882 (0.008)
4050	*LTB*	Lymphotoxin beta	−0.839 (0.013)
55647	*RAB20*	RAB20, member RAS oncogene family	−0.839 (0.028)
8351	*HIST1H3D*	Histone cluster 1, H3d	−0.809 (0.012)
1675	*CFD*	Complement factor D	−0.797 (0.009)
3134	*HLA-F*	Major histocompatibility complex, class I, F	−0.777 (0.011)
7483	*WNT9A*	Wnt family member 9A	0.758 (0.028)
6495	*SIX1*	SIX homeobox 1	−0.700 (0.027)
8092	*ALX1*	ALX homeobox 1	0.660 (0.014)
374879	*ZNF699*	Zinc finger protein 699	0.658 (0.013)
11092	*SPACA9*	Sperm acrosome associated 9	−0.651 (0.005)
2537	*IFI6*	Interferon alpha inducible protein 6	−0.642 (0.000)
241	*ALOX5AP*	Arachidonate 5-lipoxygenase activating protein	0.633 (0.001)
100288332	*NPIPA5*	Nuclear pore complex interacting protein family member A5	0.622 (0.026)

**Table 3:** List of changes in gene expression in HAECs and T2D-HAECs after exposure to 100 mGy radiation.

Entrez _ID	Gene Symbol	log_2_(fc) (*p*-Value)	
		HAECs	T2D-HAECs
51554	*ACKR4*	0.375 (0.042)	−0.333 (0.003)
28990	*ASTE1*	−0.269 (0.006)	−0.285 (0.031)
129607	*CMPK2*	0.430 (0.009)	−0.333 (0.029)
3430	*CXCL10*	0.795 (0.014)	−0.405 (0.048)
55008	*HERC6*	0.347 (0.025)	−0.340 (0.028)
64135	*IFI35*	0.403 (0.002)	−0.358 (0.032)
3434	*IFIH1*	0.367 (0.016)	−0.329 (0.015)
9636	*IFIT1*	0.427 (0.009)	−0.468 (0.020)
3437	*IFIT3*	0.297 (0.019)	−0.304 (0.040)
51056	*ISG15*	0.357 (0.021)	−0.544 (0.030)
91543	*LAP3*	0.308 (0.010)	−0.347 (0.045)
11274	*RSAD2*	0.516 (0.031)	−0.490 (0.001)
50838	*TAS2R13*	−0.559 (0.002)	−0.529 (0.009)
259295	*TAS2R20*	−0.438 (0.009)	0.497 (0.003)
3627	*USP18*	0.277 (0.004)	−0.346 (0.001)

HAECs = human aortic endothelial cells; T2D-HAECs = human aortic endothelial cells, diabetes type II.

**Table 4:** Primers used in qRT-PCR.

Gene Symbol	Forward Sequence	Reverse Sequence
*ACKR4*	CCCGCTACCTAGGAACATCA	TCTATGGCTCGGCAGAACTT
*CMPK2*	CTGAGGAGAGGTTGCAGAGG	CTGCAGGACCTTTTCTCTGG
*CXCL10*	CTGTACGCTGTACCTGCATCA	TTCTTGATGGCCTTCGATTC
*IFI35*	CCATTTTCAGTGCCCAAGAT	TTGATCGTGTGCTCCTTTTG
*IFIH1*	ACCAAATACAGGAGCCATGC	GCGATTTCCTTCTTTTGCAG
*IFIT1*	AAAAGCCCACATTTGAGGTG	GAAATTCCTGAAACCGACCA
*ISG15*	TGTCGGTGTCAGAGCTGAAG	GCCCTTGTTATTCCTCACCA
*LAP3*	TTTGCTTCTGGGCAGAACTT	CTTTGGCCACACTGAGGAAT
*RSAD2*	CTCGCCAGTGCAACTACAAA	CACCAACTTGCCCAGGTATT
*USP18*	CTGTGCCATGGAGAGTAGCA	AGGTGGATTGTCAGGGTCTG

## Data Availability

The data that support the findings of this study are available from the corresponding author upon reasonable request.

## Supplementary Materials

The following supporting information can be downloaded at: https://www.mdpi.com/article/10.3390/ijms23158577/s1.

## References

[1] Brenner D.J., Doll R., Goodhead D.T., Hall E.J., Land C.E., Little J.B., Lubin J.H., Preston D.L., Preston R.J., Puskin J.S. (2003). Cancer risks attributable to low doses of ionizing radiation: Assessing what we really know. Proc. Natl. Acad. Sci. USA.

[2] Valentin J. (2007). The 2007 Recommendations of the International Commission on Radiological Protection.

[3] Wrixon A.D. (2008). New ICRP recommendations. J. Radiol. Prot..

[4] Fazel R., Krumholz H.M., Wang Y., Ross J.S., Chen J., Ting H.H., Shah N.D., Nasir K., Einstein A.J., Nallamothu B.K. (2009). Exposure to low-dose ionizing radiation from medical imaging procedures. N. Engl. J. Med..

[5] Little M.P., Lipshultz S.E. (2015). Low dose radiation and circulatory diseases: A brief narrative review. Cardiooncology.

[6] Alexander Y., Osto E., Schmidt-Trucksass A., Shechter M., Trifunovic D., Duncker D.J., Aboyans V., Back M., Badimon L., Cosentino F. (2021). Endothelial function in cardiovascular medicine: A consensus paper of the European Society of Cardiology Working Groups on Atherosclerosis and Vascular Biology, Aorta and Peripheral Vascular Diseases, Coronary Pathophysiology and Microcirculation, and Thrombosis. Cardiovasc. Res..

[7] Leon B.M., Maddox T.M. (2015). Diabetes and cardiovascular disease: Epidemiology, biological mechanisms, treatment recommendations and future research. World J. Diabetes.

[8] Naveed A., Farrukh L., Sana M.K., Naveed B., Randhawa F.A. (2020). Pharmacological Primary Prevention of Diabetes Mellitus Type II: A Narrative Review. Cureus.

[9] Teinturier C., Tournade M.F., Caillat-Zucman S., Boitard C., Amoura Z., Bougneres P.F., Timsit J. (1995). Diabetes mellitus after abdominal radiation therapy. Lancet.

[10] Meacham L.R., Sklar C.A., Li S., Liu Q., Gimpel N., Yasui Y., Whitton J.A., Stovall M., Robison L.L., Oeffinger K.C. (2009). Diabetes mellitus in long-term survivors of childhood cancer. Increased risk associated with radiation therapy: A report for the childhood cancer survivor study. Arch. Intern. Med..

[11] Rose S.R., Horne V.E., Howell J., Lawson S.A., Rutter M.M., Trotman G.E., Corathers S.D. (2016). Late endocrine effects of childhood cancer. Nat. Rev. Endocrinol..

[12] Friedman D.N., Tonorezos E.S., Cohen P. (2019). Diabetes and Metabolic Syndrome in Survivors of Childhood Cancer. Horm. Res. Paediatr..

[13] Poonsombudlert K., Limpruttidham N. (2019). Total Body Irradiation and Risk of Diabetes Mellitus; A Meta-Analysis. Asian Pac. J. Cancer Prev..

[14] Barnea D., Raghunathan N., Friedman D.N., Tonorezos E.S. (2015). Obesity and Metabolic Disease After Childhood Cancer. Oncology.

[15] Meacham L.R., Chow E.J., Ness K.K., Kamdar K.Y., Chen Y., Yasui Y., Oeffinger K.C., Sklar C.A., Robison L.L., Mertens A.C. (2010). Cardiovascular risk factors in adult survivors of pediatric cancer—A report from the childhood cancer survivor study. Cancer Epidemiol. Biomark. Prev..

[16] Tatsukawa Y., Cordova K., Yamada M., Ohishi W., Imaizumi M., Hida A., Sposto R., Sakata R., Fujiwara S., Nakanishi S. (2022). Incidence of Diabetes in the Atomic Bomb Survivors: 1969–2015. J. Clin. Endocrinol. Metab..

[17] Zhang F., Lin X., Yu L., Li W., Qian D., Cheng P., He L., Yang H., Zhang C. (2016). Low-dose radiation prevents type 1 diabetes-induced cardiomyopathy via activation of AKT mediated anti-apoptotic and anti-oxidant effects. J. Cell Mol. Med..

[18] Zhang C., Jin S., Guo W., Li C., Li X., Rane M.J., Wang G., Cai L. (2011). Attenuation of diabetes-induced cardiac inflammation and pathological remodeling by low-dose radiation. Radiat. Res..

[19] Wang G.J., Li X.K., Sakai K., Lu C. (2008). Low-dose radiation and its clinical implications: Diabetes. Hum. Exp. Toxicol..

[20] Hadi H.A., Suwaidi J.A. (2007). Endothelial dysfunction in diabetes mellitus. Vasc. Health Risk Manag..

[21] Cai S., Khoo J., Mussa S., Alp N.J., Channon K.M. (2005). Endothelial nitric oxide synthase dysfunction in diabetic mice: Importance of tetrahydrobiopterin in eNOS dimerisation. Diabetologia.

[22] Cutiongco M.F.A., Chua B.M.X., Neo D.J.H., Rizwan M., Yim E.K.F. (2018). Functional differences between healthy and diabetic endothelial cells on topographical cues. Biomaterials.

[23] Zhu D.D., Tang R.N., Lv L.L., Wen Y., Liu H., Zhang X.L., Ma K.L., Liu B.C. (2016). Interleukin-1beta mediates high glucose induced phenotypic transition in human aortic endothelial cells. Cardiovasc. Diabetol..

[24] Chen J., Huang X., Halicka D., Brodsky S., Avram A., Eskander J., Bloomgarden N.A., Darzynkiewicz Z., Goligorsky M.S. (2006). Contribution of p16INK4a and p21CIP1 pathways to induction of premature senescence of human endothelial cells: Permissive role of p53. Am. J. Physiol. Heart Circ. Physiol..

[25] Nemet A.Y., Vinker S., Levartovsky S., Kaiserman I. (2010). Is cataract associated with cardiovascular morbidity?. Eye.

[26] Shimizu Y., Kodama K., Nishi N., Kasagi F., Suyama A., Soda M., Grant E.J., Sugiyama H., Sakata R., Moriwaki H. (2010). Radiation exposure and circulatory disease risk: Hiroshima and Nagasaki atomic bomb survivor data, 1950–2003. BMJ.

[27] Kreuzer M., Auvinen A., Cardis E., Hall J., Jourdain J.R., Laurier D., Little M.P., Peters A., Raj K., Russell N.S. (2015). Low-dose ionising radiation and cardiovascular diseases--Strategies for molecular epidemiological studies in Europe. Mutat. Res. Rev. Mutat. Res..

[28] Dong G., Qu L., Gong X., Pang B., Yan W., Wei J. (2019). Effect of Social Factors and the Natural Environment on the Etiology and Pathogenesis of Diabetes Mellitus. Int. J. Endocrinol..

[29] Wang Q.Q., Yin G., Huang J.R., Xi S.J., Qian F., Lee R.X., Peng X.C., Tang F.R. (2021). Ionizing Radiation-Induced Brain Cell Aging and the Potential Underlying Molecular Mechanisms. Cells.

[30] Pernow J., Jung C. (2016). The Emerging Role of Arginase in Endothelial Dysfunction in Diabetes. Curr. Vasc. Pharmacol..

[31] Vieira Dias J., Gloaguen C., Kereselidze D., Manens L., Tack K., Ebrahimian T.G. (2018). Gamma Low-Dose-Rate Ionizing Radiation Stimulates Adaptive Functional and Molecular Response in Human Aortic Endothelial Cells in a Threshold-, Dose-, and Dose Rate-Dependent Manner. Dose Response.

[32] Takehara Y., Yamaoka K., Hiraki Y., Yoshioka T., Utsumi K. (1995). Protection against alloxan diabetes by low-dose 60Co gamma irradiation before alloxan administration. Physiol. Chem. Phys. Med. NMR.

[33] Rombouts C., Aerts A., Beck M., De Vos W.H., Van Oostveldt P., Benotmane M.A., Baatout S. (2013). Differential response to acute low dose radiation in primary and immortalized endothelial cells. Int. J. Radiat. Biol..

[34] De Rosa S., Arcidiacono B., Chiefari E., Brunetti A., Indolfi C., Foti D.P. (2018). Type 2 Diabetes Mellitus and Cardiovascular Disease: Genetic and Epigenetic Links. Front. Endocrinol..

[35] Boshuizen M.C., de Winther M.P. (2015). Interferons as Essential Modulators of Atherosclerosis. Arter. Thromb. Vasc. Biol..

[36] Wu X.M., Zhang J., Li P.W., Hu Y.W., Cao L., Ouyang S., Bi Y.H., Nie P., Chang M.X. (2020). NOD1 Promotes Antiviral Signaling by Binding Viral RNA and Regulating the Interaction of MDA5 and MAVS. J. Immunol..

[37] Liu G., Lee J.H., Parker Z.M., Acharya D., Chiang J.J., van Gent M., Riedl W., Davis-Gardner M.E., Wies E., Chiang C. (2021). ISG15-dependent activation of the sensor MDA5 is antagonized by the SARS-CoV-2 papain-like protease to evade host innate immunity. Nat. Microbiol..

[38] Filippi C.M., von Herrath M.G. (2008). Viral trigger for type 1 diabetes: Pros and cons. Diabetes.

[39] Chen C., Tian J., He Z., Xiong W., He Y., Liu S. (2021). Identified Three Interferon Induced Proteins as Novel Biomarkers of Human Ischemic Cardiomyopathy. Int. J. Mol. Sci..

[40] Jian D., Wang W., Zhou X., Jia Z., Wang J., Yang M., Zhao W., Jiang Z., Hu X., Zhu J. (2018). Interferon-induced protein 35 inhibits endothelial cell proliferation, migration and re-endothelialization of injured arteries by inhibiting the nuclear factor-kappa B pathway. Acta Physiol..

[41] Malakhova O.A., Kim K.I., Luo J.K., Zou W., Kumar K.G., Fuchs S.Y., Shuai K., Zhang D.E. (2006). UBP43 is a novel regulator of interferon signaling independent of its ISG15 isopeptidase activity. EMBO J..

[42] Rahnefeld A., Klingel K., Schuermann A., Diny N.L., Althof N., Lindner A., Bleienheuft P., Savvatis K., Respondek D., Opitz E. (2014). Ubiquitin-like protein ISG15 (interferon-stimulated gene of 15 kDa) in host defense against heart failure in a mouse model of virus-induced cardiomyopathy. Circulation.

[43] Lai J.H., Hung L.F., Huang C.Y., Wu D.W., Wu C.H., Ho L.J. (2021). Mitochondrial protein CMPK2 regulates IFN alpha-enhanced foam cell formation, potentially contributing to premature atherosclerosis in SLE. Arthritis Res. Ther..

[44] Chmielewski S., Piaszyk-Borychowska A., Wesoly J., Bluyssen H.A. (2016). STAT1 and IRF8 in Vascular Inflammation and Cardiovascular Disease: Diagnostic and Therapeutic Potential. Int. Rev. Immunol..

[45] Groblewska M., Litman-Zawadzka A., Mroczko B. (2020). The Role of Selected Chemokines and Their Receptors in the Development of Gliomas. Int. J. Mol. Sci..

[46] Gosling J., Dairaghi D.J., Wang Y., Hanley M., Talbot D., Miao Z., Schall T.J. (2000). Cutting edge: Identification of a novel chemokine receptor that binds dendritic cell- and T cell-active chemokines including ELC, SLC, and TECK. J. Immunol..

[47] Watts A.O., Verkaar F., van der Lee M.M., Timmerman C.A., Kuijer M., van Offenbeek J., van Lith L.H., Smit M.J., Leurs R., Zaman G.J. (2013). beta-Arrestin recruitment and G protein signaling by the atypical human chemokine decoy receptor CCX-CKR. J. Biol. Chem..

[48] Vinet J., van Zwam M., Dijkstra I.M., Brouwer N., van Weering H.R., Watts A., Meijer M., Fokkens M.R., Kannan V., Verzijl D. (2013). Inhibition of CXCR3-mediated chemotaxis by the human chemokine receptor-like protein CCX-CKR. Br. J. Pharmacol..

[49] Patel P.A., Tilley D.G., Rockman H.A. (2008). Beta-arrestin-mediated signaling in the heart. Circ. J..

[50] Feng L., Chen Y., Xu K., Li Y., Riaz F., Lu K., Chen Q., Du X., Wu L., Cao D. (2022). Cholesterol-induced leucine aminopeptidase 3 (LAP3) upregulation inhibits cell autophagy in pathogenesis of NAFLD. Aging.

[51] Lonardo A., Nascimbeni F., Mantovani A., Targher G. (2018). Hypertension, diabetes, atherosclerosis and NASH: Cause or consequence?. J. Hepatol..

[52] Kaushik N., Kim M.J., Kim R.K., Kumar Kaushik N., Seong K.M., Nam S.Y., Lee S.J. (2017). Low-dose radiation decreases tumor progression via the inhibition of the JAK1/STAT3 signaling axis in breast cancer cell lines. Sci. Rep..

[53] Langmead B., Salzberg S.L. (2012). Fast gapped-read alignment with Bowtie 2. Nat. Methods.

